# Prevalence of Potential Drug-Drug Interactions Involving Antiretroviral Drugs in a Large Kenyan Cohort

**DOI:** 10.1371/journal.pone.0016800

**Published:** 2011-02-23

**Authors:** Gabriel Kigen, Sylvester Kimaiyo, Winstone Nyandiko, Brian Faragher, Edwin Sang, Beatrice Jakait, Andrew Owen, David Back, Sara Gibbons, Kay Seden, Saye H. Khoo, on behalf of the USAID-Academic Model for Prevention Treatment of HIV/AIDS

**Affiliations:** 1 Department of Pharmacology and Toxicology, Moi University School of Medicine, Eldoret, Kenya; 2 Department of Pharmacology, Institute of Translational Medicine, The University of Liverpool, Liverpool, United Kingdom; 3 USAID-Academic Model Providing Access to Healthcare (AMPATH), Moi University School of Medicine, Eldoret, Kenya; 4 Liverpool School of Tropical Medicine, Liverpool, United Kingdom; 5 NIHR Biomedical Research Centre for Microbial Diseases, Royal Liverpool University Hospital, Liverpool, United Kingdom; University of Cape Town, South Africa

## Abstract

**Background:**

Clinically significant drug-drug interactions (CSDIs) involving antiretrovirals are frequent and under-recognized in developed countries, but data are lacking for developing countries.

**Methodology and Principal Findings:**

To investigate the prevalence of CSDIs between antiretrovirals and coadministered drugs, we surveyed prescriptions dispensed in a large HIV clinic in Kenya. Of 1040 consecutive patients screened, 996 were eligible for inclusion. CSDIs were defined as ‘major’ (capable of causing severe or permanent damage, contraindicated, avoid or not recommended by the manufacturer, or requiring dose modification) or ‘moderate’ (manufacturers advise caution, or close monitoring, or capable of causing clinical deterioration). A total of 334 patients (33.5%) were at risk for a CSDI, potentially lowering antiretroviral drug concentrations in 120 (12%) patients. Major interactions most frequently involved rifampicin (12.4%, mostly with efavirenz) and azoles (2.7%) whereas moderate interactions were frequently azoles (13%), steroids (11%), and antimalarials (3%). Multivariable analyses suggested that patients at risk for CSDIs had lower CD4 counts (P = 0.006) and baseline weight (P = 0.023) and WHO Stage 3 or 4 disease (P≤0.007). Risk for CSDIs was not associated with particular regimens, although only 116 (11.6%) patients were receiving WHO second line regimens.

**Conclusions:**

One in three patients receiving antiretrovirals in our programme were at risk of CSDIs. Strategies need to be urgently developed to avoid important drug interactions, to identify early markers of toxicity and to manage unavoidable interactions safely in order to reduce risk of harm, and to maximize the effectiveness of mass antiretroviral deployment in Africa.

## Introduction

The introduction of combination antiretroviral therapy has remarkably improved survival of HIV infected persons [1]. Of the five main classes of antiretrovirals, only nucleoside reverse transcriptase inhibitor (NRTIs), non-nucleoside reverse transcriptase inhibitor (NNRTIs) and protease inhibitors (PIs) are widely available for treatment of HIV in sub-Saharan Africa.

NNRTIs and PIs undergo biotransformation by cytochrome P-450 (CYP) enzymes, thus making them prone to clinically significant drug interactions (CSDI) when combined with other drugs metabolised via the same pathway. In addition they interact with other medications, acting as either inducers or inhibitors of CYP enzymes. Further, PIs are substrates and/or inhibitors of drug transporters such as P-glycoprotein which may result in pharmacokinetic drug interactions [2]–[4]. Although most NRTIs are renally excreted, there remains a potential for drug interactions [2], [3]. Managing drug-drug interactions is one of the major challenges in the optimisation of HIV therapy [5], [6]. CSDIs have previously been reported to be prevalent in developed countries (affecting 20–41% patients) [7]–[11], but data from developing countries are lacking. HIV infected individuals in Africa often present late, with acute opportunistic infections and other AIDS-associated conditions which require multiple other medications thus increasing the potential for CSDIs.

USAID-AMPATH is a partnership between Indiana University School of Medicine and Moi University School of Medicine (Kenya) and is one of Africa's largest antiretroviral programs. It is based at the Moi Teaching and Referral Hospital. During the period of study AMPATH cared for more than 55,000 HIV infected adults and children, with nearly one-half of all patients on antiretroviral drugs, and enrolment into the program was increasing by 2,000 patients per month [12]. In this prospective observational survey, we investigated the frequency of CSDIs in follow up prescriptions for 1000 consecutive patients enrolled into the AMPATH programme.

## Methods

### Study design

The study was approved by the institutional research and ethics committee of Moi Teaching and Referral Hospital and Moi University School of Medicine. Since we were utilising only anonymised data from the AMPATH and pharmacy databases, the ethics committee allowed the study to be conducted without individual patient consent. In addition to prescription data (which were prospectively recorded by the AMPATH programme), we also collected patient demographic data at enrolment. In order to confirm that antiretrovirals were dispensed and to record as completely as possible all co-medications prescribed, we validated all clinical records against AMPATH pharmacy records. Inclusion criteria for this study were: HIV seropositive attending for care at Moi Teaching and Referral Hospital, age >16 years, receiving antiretroviral therapy. For this study, we screened the first 1040 consecutive patients attending from 4^th^ January 2006, with follow up over a 23 month period until 19^th^ November 2007. Details of age, gender, baseline weight, WHO stage, baseline CD4 count and weight (if measured within 6 months of commencing antiretroviral therapy) and CD4 count and weight measurements nearest to end of study period, together with all antiretrovirals and concomitant medications were recorded. Under Kenyan National AIDS and STI Control Programme (NASCOP) guidelines, first-line antiretrovirals were defined as stavudine (d4T) or zidovudine (ZDV) plus lamivudine (3TC) plus nevirapine (NVP) or efavirenz (EFV), and substitution with tenofovir (TDF), abacavir (ABC) or didanosine (ddI) was allowed for toxicity. Second line included any of these agents in combination with the protease inhibitors indinavir (IDV), lopinavir/ritonavir (LPVr), or nelfinavir (NFV). The guidelines for entry into the antiretroviral programme were: i) WHO Stage 1 or 2 HIV disease if CD4 count is <200 cells/mm^3^, or ii) WHO Stage 3 disease if CD4 is <350 cells/mm^3^, or iii) WHO Stage 4 disease, irrespective of the CD4 cell count [13]. All the coprescribed drug pairs were screened for potential for CSDIs using the Liverpool HIV Pharmacology Group website (www.hiv-druginteractions.org) [14], accessed between January to August 2008. This website comprises a comprehensive database of ∼5000 drug-interaction pairs, and uses a ‘traffic lights’ system to flag up potential interactions. In order to avoid ‘overcalling’ the clinical significance of drug interactions, all interactions which flagged up as red or amber were further scrutinised, and the quality of evidence underpinning these recommendations assessed using criteria derived from the GRADE system (http://www.hiv-druginteractions.org/documents/QualityOfEvidence.pdf) [15].

### Classification of potential CSDIs

We searched the existing literature but could find no universally accepted system for classifying severity of interactions. Some publications have utilized a classification system by Tatro [16], which grades severity according to illness or laboratory abnormalities caused by that interaction, and the potential consequences of that toxicity (e.g. hospitalisation). We took the view that such a classification was not appropriate to our study setting, since: i) potentially serious adverse effects may be sub-clinical, delayed or indirect with HIV therapy (such as low plasma concentrations leading to rebound, resistance and loss of future therapeutic options in a setting where access to second or third line regimens is limited), ii) the threshold for admitting patients into hospital is considerably different in Africa compared with developed countries, and iii) clinical and laboratory monitoring is limited in resource poor settings. We therefore utilised a modified version of the Tatro severity classification as follows:

#### Major

Either potentially life-threatening, or capable of causing permanent damage; or associated with significant clinical toxicity (i.e. hospitalisation, extended hospital admission, additional treatment); or ‘contraindicated’, ‘avoid’ or ‘not recommended’ in the manufacturer's SPC (Summary of Product Characteristics; accessed from the Electronic Medicines Compendium, emc.medicines.org.uk between January to August 2008); or requiring dose modification of at least one of the drugs required in all/majority of the patients.

#### Moderate

Not a major interaction, but either capable of causing deterioration in clinical status, or where the manufacturer's SPC advises ‘caution’, or ‘close monitoring’ for toxicity or therapeutic failure.

“Major” or “moderate” interactions were classified as “clinically significant”. We classified the remainder of patients as either having no identified interactions or else minor interactions which were defined as “not a major or moderate interaction, but having usually mild consequences which may be bothersome but should not significantly affect the therapeutic outcome without requiring additional treatment.’’ Interactions relating solely to overlapping toxicities, or between co-administered antiretrovirals (e.g. PI boosting), or involving topical applications were excluded. In addition, we excluded from our analysis potential interactions for which controlled data suggested limited clinical significance (e.g. lamivudine plus cotrimoxazole).

### Statistical analysis

CD4 data were positively skewed so were log-transformed to improve approximation to Normality. In order to characterise the relationship between CD4 count and risk of drug interactions, the prevalence of CSDIs (95% CI) was compared across CD4 deciles. Patients were grouped according to whether they had a CSDI versus no significant interaction. Differences in age and baseline weight were assessed by Mann-Whitney U test, and in gender, WHO Stage and drug regimen (first or second line) were assessed by Fisher's exact test. The prevalence of CSDIs across individual NRTIs, NNRTIs and PIs were compared by Fisher's exact test. CD4 data were missing for 31 (3%) patients, weight for 11 (1%), age for 106 (11%) patients and WHO Stage for 10 (1%) patients. We therefore undertook multiple missing values imputation over 100 iterations prior to performing univariable and multivariable analyses. Multivariable logistic regression was performed (with the following covariates forced into the model: age, gender, baseline weight, drug regimen, WHO Stage and log CD4 count) using the PASW (SPPS version 17; SPSS Inc, Chicago, USA) statistical package.

## Results

Of 1040 patients screened, 40 patients were excluded as they were aged <13 years, and a further 4 patients who discontinued antiretrovirals during an acute opportunistic infection were also excluded. A total of 996 patients were included in the final analysis, comprising 346 (35%) male and 650 (65%) females aged between 21–68 years (mean 39 years), mean body weight at baseline of 58.1 kg (range 20–99 kg), and follow up for 1–22 months (median 15 months; total of 15,060 person-months). Baseline CD4 counts were available for 965 patients with a geometric mean count of 108 (range 0–1137) cells/mm^3^. During follow up, the average change in CD4 count (95% CI) recorded for 742 patients was +125 (112–138) cells and weight recorded for 720 patients was +3.1 (2.6–3.7) kg.

A total of 880 patients (88.4%) were on first-line antiretroviral therapy, and 116 (11.6%) patients were on second line treatment. The use of 1^st^ line regimens were as follows: d4T/3TC/NVP in 504 patients (50.6%), d4T/3TC/EFV in 194 patients (19.5%), 3TC/ZDV/EFV in 44 patients (4.4%), ZDV/3TC/NVP in 128 patients (12.9%), ABC/3TC/NVP in 3 patients (0.3%), TDF/3TC/EFV in 3 patients (0.3%) and TDF/3TC/NVP in 4 patients (0.4%). Use of 2^nd^ line treatments were as follows: 3TC/ZDV/NFV in 34 patients (3.4%), ABC/ddI/LPVr in 28 patients (2.8%), ABC/ZDV/LPVr in 16 patients (1.6%), d4T/3TC/LPVr in 2 patients (0.2%), NFV/3TC/d4T in 9 patients (0.9%), ZDV/3TC/LPVr in 12 patients (1.2%), ZDV/ddI/LPVr in 14 patients (1.4%) and ABC/3TC/LPVr in 1 patient (0.1%). Use of individual drugs was as follows: d4T 709 (71.2%), 3TC 938 (94.2%), ZDV 248 (24.9%), NVP 639 (64.2%), EFV 241 (24.2%), TDF 7 (0.7%), LPVr 70 (7.0%), NFV 43(4.3%), ddI 42 (4.2%) and ABC 48 (4.8%). All antiretrovirals were prescribed at standard doses, regardless of whether a CSDI was present or not.

Risk for clinically significant interactions was identified in 334 patients (33.5%) with major interactions in 147 patients (14.8%), predominantly involving rifampicin (124 patients; 12.4%) and azoles (27 patients; 2.7%) (Table 1). Potential for moderate interactions were identified in 230 patients (23.1%), involving azoles in 129 patients (13%), steroids in 106 patients (10.6%) and antimalarials in 29 patients (2.9%). No, or minor, drug interactions were recorded in 662 patients (66.5%). Of the patients with major/moderate interactions, 251 patients had one major/moderate interaction recorded while 83 patients had more than one, with 70 patients having two interactions, 11 patients three interactions and two patients having four interactions.

**Table 1:** Prevalence and Nature of Clinically Significant Drug Interactions in Kenya.

Co-prescribed drug pairs	No. of interactions(% of 432 CSDIs*)
**Major interactions**	
LPVr + artemether/lumefantrine	1 (0.2)
LPVr + fluoxetine	2 (0.5)
LPVr + rifampicin	3 (0.7)
Efavirenz + rifampicin	76 (17.6)
Nelfinavir + lansoprazole	40.9)
Nelfinavir + omeprazole	4 (0.9)
Nevirapine + ketoconazole	27 (6.3)
Nevirapine + rifampicin	45 (10.4)
**Moderate interactions**	
LPVr + ketoconazole	2 (0.5)
Efavirenz + ketoconazole	10 (2.3)
Efavirenz + artemether/lumefantrine	5 (1.2)
Nevirapine + artemether/lumefantrine	24 (5.6)
Nevirapine + fluconazole	97 (22.5)
Nevirapine + prednisone	106 (24.5)
Zidovudine + dapsone	1 (0.2)
Zidovudine + fluconazole	20 (4.6)
Zidovudine + rifampicin	5 (1.2)

*occurring in 334 individuals.

Of the 432 CSDIs in 334 patients, 137 interactions occurring in 120 patients (35.9%) could potentially have resulted in decreased antiretroviral exposure. Examples of these were: lopinavir/ritonavir and rifampicin, nevirapine and rifampicin, nelfinavir and omeprazole, nelfinavir and lansoprazole, and zidovudine and rifampicin. In contrast to other studies [9], [11], patients receiving PIs were not more likely to have a CSDI. Excluding patients on nelfinavir showed a comparable prevalence of CSDIs between patients receiving lopinavir/ritonavir (31.5%), nevirapine (34.3%) and efavirenz (36.5%). However, this probably masks considerable differences in underlying patient characteristics - for example, co-administration of rifampicin was more frequent with efavirenz (76/241; 32%), compared to nevirapine (45/639; 7%) or lopinavir/ritonavir (3/70; 2.6%. p<0.0001). Thus choice of regimen was driven to a large degree by the need to manage simultaneous tuberculosis (TB) and HIV treatment. Efavirenz with rifampicin was in fact the most frequently encountered major interaction (Table 1), and since this was prescribed in line with best practice in HIV/TB co-infected patients, it could be argued that this was an interaction which was being optimally managed. Exclusion of the 40 patients in whom efavirenz plus rifampicin was the sole CSDI yielded 294 patients (29.5%) with CSDIs.

Univariate analyses (Table 2) suggested that patients at risk of CSDIs were significantly more likely to have a lower mean baseline weight (56.0 vs 59.1 kg; p<0.001), lower CD4 count (geometric mean 86 vs 120 cells/mm^3^; p<0.001) and more advanced disease at WHO Stage 3 (48.8% vs 34.1%; p<0.001) or Stage 4 (10.9% vs 7.0%; p<0.001). A weaker association was observed between risk of CDSI and use of second line therapy (p = 0.014). This may have been confounded by avoidance of PIs in patients with tuberculosis, and the association was lost in multivariate analyses. No significant association was observed between risk for CDSIs and change in CD4 count or body weight, and these covariates were not included in further analyses.

**Table 2:** Factors Associated with Risk for Developing a Clinically Significant Drug Interaction.

Variable		Clinically significant drug interaction		Odds ratio (95% CI) [p value]*	
		no	yes	unadjusted	adjusted
Sample size		662	334		
Sex: male	n (%)	239 (36.1)	107 (32.0)	---	---
female	n (%)	423 (63.9)	227 (68.0)	1.199 (0.902–1.592 [0.203]	1.506 (1.097–2.065) [0.010]
Age (y)	mean (sd)	38.9 (9.2)	39.5 (8.6)	1.009 (0.993–1.025) [0.289]	1.010 (0.992–1.028) [0.243]
Weight (kg)	mean (sd)	59.1 (10.7)	56.0 (10.5)	0.971 (0.958–0.985) [<0.001]	0.984 (0.970–0.998) [0.023]
CD4 (log units)	mean (sd)	4.79 (1.10)	4.46 (1.21)	0.777 (0.690–0.874) [<0.001]	0.839 (0.739–0.954) [0.006]
WHO Stage: 1	n (%)	277 (42.2)	86 (26.1)	---	---
2	n (%)	109 (16.6)	47 (14.2)	1.386 (0.903–2.125) [0.127]	1.203 (0.776–1.865) [0.400]
3	n (%)	224 (34.1)	161 (48.8)	2.301 (1.667–3.174) [<0.001]	1.886 (1.327–2.683) [<0.001]
4	n (%)	46 (7.0)	36 (10.9)	2.507 (1.508–4.166) [<0.001]	2.085 (1.213–3.586) [0.007]
Regimen: 1^st^ line	n (%)	573 (86.6)	307 (91.9)	---	---
2^nd^ line	n (%)	89 (13.4)	27 (8.1)	0.566 (0.360–0.890) [0.014]	0.742 (0.457–1.203) [0.218]

Summary statistics are based on original data (ignoring the missing observations). However, both the unadjusted (univariate) and adjusted (bivariate) odds ratios (and their 95% confidence intervals) were computed after a multiple imputation analysis (of 100 iterations) to replace the missing observations for age, CD4 count, weight and WHO Stage. The odds ratios were computed using logistic regression models (with all variables forced into the model in the adjusted/multivariate analysis).

*: missing observations replaced using multiple imputation (100 iterations).

Amongst NRTIs, no single drug was significantly associated with increased risk for CSDIs (Table 3). As numbers of patients receiving tenofovir were low, Fisher's test for significance was carried out with and without these patients. There was also no significant difference in risk for CSDIs between nevirapine and efavirenz. Patients receiving nelfinavir were significantly less likely to be at risk for a CSDI than those on lopinavir/ritonavir but numbers were small, and the relevance of this finding is limited, given that nelfinavir is no longer prescribed in most countries.

**Table 3:** Risk of Clinically Significant Drug Interaction by Individual Drug.

Antiretroviral drug	Total no. of patients	Clinically significant drug interactionN (%; 95% CI)	p value‡
**NRTIs**			
Abacavir (ABC)	48	14 (29.2%; 17.0–44.1)	
Didanosine (ddI)	42	18 (42.9%; 27.7–59.0)	all NRTIs
Lamivudine (3TC)	938	313 (33.4%; 30.4–36.5)	0.547
Stavudine (d4T)	709	239 (33.7%; 30.2–37.3)	
Zidovudine (ZDV)	248	80 (32.3%; 26.5–38.5)	excluding TDF
Tenofovir (TDF)	7	4 (57.1%; 18.4–90.1)	0.694
**NNRTIs**			
Efavirenz (EFV)	241	88 (36.5%; 30.4 – 42.9)	0.579
Nevirapine (NVP)	639	219 (34.3%; 30.6–38.1)	
**PIs**			
Lopinavir (LPVr)	73	23 (31.5%; 21.1–43.4)	0.006
Nelfinavir (NFV)	43	4 (9.3%; 2.6–22.1)	

***‡: Fisher exact test.***

**Figure 1:**
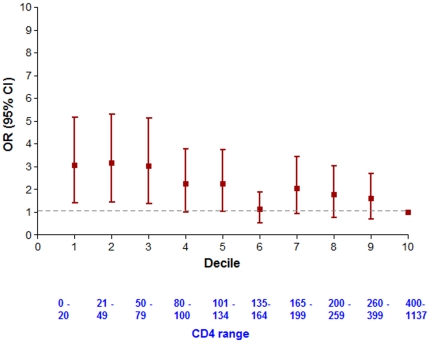
Odds Ratios for Clinically Significant Drug Interaction by CD4 Count Decile.

Multivariable logistic regression (Table 2) revealed that the risk for CSDIs was significantly associated with female gender (68.0% vs 63.9%; OR 1.506 [95% CI 1.097–2.065]; p = 0.010), low baseline weight (OR 0.984 [0.970–0.998]; p = 0.023), low baseline CD4 count (OR 0.839 [0.739–0.954]; p = 0.006) and disease stage at WHO Stage 3 (OR 1.886 [1.327–2.683]; p<0.001) or Stage 4 (OR 2.085 [1.213–3.586]; p = 0.007). Analysis of the relationship between CSDIs and CD4 decile (Figure 1) showed that the prevalence of CSDIs increased with decreasing CD4 counts.

## Discussion

Drug-drug interactions are one of the commonest causes of medication error in developed countries, and antiretrovirals among the most therapeutically risky drugs for clinically significant drug interactions. Studies in the Netherlands and New York involving 115 and 550 patients suggest a prevalence of 20–25% CSDIs [7], [8]. A second study in New York involving 153 patients reported a prevalence of 41.2% [9]. Two recent studies conducted in Liverpool (159 patients) and Switzerland (1497 patients) reported a prevalence of CSDIs (including drug-drug interactions between antiretroviral agents) of 26.3% and 40% respectively [10], [11]. Although definitions differed, four out of five of these studies utilised the Liverpool Drug Interactions website to screen for interactions. There have been no such studies in resource-limited settings where risk is arguably increased as a result of less laboratory monitoring, high rates of background illness (which may result in adverse effects being missed), lack of affordable alternative treatments, use of fixed dose combinations (that offer less flexibility for managing interactions) and lack of pharmacovigilance data. In addition, there is a higher cost of treatment failure in these settings, since options are limited compared with developed countries. The purpose of our study was to investigate the potential for CSDIs between antiretrovirals and co-administered drugs in a large outpatient cohort in Kenya.

In Africa, the concurrent epidemics of HIV, TB and malaria to a large degree make CSDIs unavoidable. We observed that risk for CSDIs was prevalent in the patients studied, affecting one in three patients. Of particular concern, these interactions could have resulted in lowering of plasma concentrations of antiretrovirals (thus increasing the risk of HIV treatment failure) in over a third of patients with CSDIs. Although the repertoire of available drugs may be more limited, risk of adverse outcome resulting from CSDIs is arguably higher in resource-poor settings due to lack of intensive laboratory monitoring, presence of overlapping syndromes such as fever (which may confound the correct identification of adverse events), late presentation of HIV, high background of other illness and use of traditional medicines and antimalarials in the community. Patients with advanced (WHO Stage 3 or 4) disease were approximately twice as likely to be at risk of a CSDI. Risk was increased with low CD4 count, but there were also weaker correlations with low baseline weight, and female gender. Furthermore, the use of fixed dose combinations of antiretrovirals gives little scope for dose-modification as a strategy for managing these interactions.

Most of the major interactions involved interactions between antiretrovirals and rifampicin in patients who were being treated for TB. Despite the growing number of clinical trials assessing novel TB drugs, there is still no credible alternative to rifamycin-based therapy, and this remained the predominant cause of CSDIs in our study. Use of nevirapine with rifampicin remains prevalent in sub-Saharan Africa. Pending results from comparative trials (without nevirapine lead-in dosing), efavirenz-based regimens are preferred since they have been shown to be effective, and less affected by rifampicin. Rifampicin was reported to decrease nevirapine AUC concentrations by 58% [17], [18] and efavirenz AUC by 26% [19], but virological outcomes in people receiving standard dose efavirenz and rifampicin are comparable with patients commencing antiretrovirals without TB [20].

In patients with NNRTI resistance, treatment choices in resource poor settings are difficult, balancing an increased risk of HIV treatment failure against unaffordable drug costs. NRTI-only regimens may be an option for the duration of rifampicin therapy for those in whom prior resistance is unlikely. Double-dose LPVr has been proposed but not formally evaluated for adult patients (drug exposure in very young children was found to be inadequate [21], [22]). Added ritonavir boosting may also be an option in some health settings. Newer drugs such as raltegravir and maraviroc are not affordable options for most treatment programmes in sub-Saharan Africa. Substituting rifampicin with rifabutin is effective in predominantly HIV-negative or untested cohorts [23] but is also currently unaffordable for many health care settings. The optimal dosing of rifabutin with LPVr is currently a subject of debate [24].

The widespread use of azoles, (either as treatment for *Candida* infections, or prophylaxis against cryptococcal disease) also accounted for a significant number of interactions. Interactions involving nevirapine and fluconazole were identified in 97 patients (9.7%), while nevirapine and ketoconazole were recorded for 27 patients (2.7%). Nevirapine has been reported to decrease ketoconazole concentrations by 72% upon coadministration [25] and the combination is contraindicated in the manufacturer's SPC. We have recently reported that co-administration of fluconazole and nevirapine resulted in a 33% increase in nevirapine AUC_0–8h_ compared to when nevirapine was administered alone [26] and therefore there is potential for toxicity.

Artemether-combination therapies have replaced sulfadoxine/pyrimethamine as first line antimalarials, and have the potential for clinically relevant pharmacokinetic interactions and toxicity with HIV PIs and NNRTIs. The AUC of lumefantrine increased by 193% in a study when lopinavir/ritonavir was coadministered with artemether/lumefantrine, since lumefantrine is extensively metabolized by CYP3A4 and PIs inhibit CYP3A4 [27], and the manufacturer's SPC advises that the combination is contraindicated. Of the other interactions, proton pump inhibitors were important; mainly with nelfinavir, a PI no longer in widespread use in developed countries but may become an increasing problem if atazanavir is used in second line regimens.

There are several limitations to our study. It is important to note that we studied the potential for CSDIs, and did not monitor for adverse outcomes arising from these interactions. Specifically our lack of ability to closely monitor viral load and liver biochemistry rendered us unable to assess the impact of adverse interactions. We did not observe any relationship between risk for CSDIs and response to therapy (as measured by CD4 count and weight gain) but these measures of efficacy are insensitive, do not correlate tightly with HIV viral load, and were missing in over a quarter of our patients. Current WHO guidance and national policy in Kenya recommends the use of efavirenz (without weight-based dose modifications) when using rifampicin for TB co-infection. Thus, these drugs were prescribed in accordance with existing best practice even though they would have appeared as major interactions in our series (excluding these patients did not significantly affect the prevalence of patients at risk of CSDIs). We did not actively seek information on the use of antimalarials, or herbal or traditional medicines in the community, and the use of oral/injectable contraceptives was almost certainly incompletely recorded. We also did not include drug interactions between the non-HIV medications. Finally, we did not ascertain whether the patients were receiving treatment elsewhere other than the HIV clinic. Our findings may therefore represent an underestimation of the true incidence of potentially significant drug interactions in this cohort of patients.

We observed a prevalence of 11.6% patients on second line (PI-based) regimens which is comparable to a frequency of switch to PI-based second-line regimens of 11.4% at 36 months in 5484 children in the IeDEA cohort in South Africa [28], 1.7% at 2 years in 1,045 adults in the Home-based AIDS Care cohort from Eastern Uganda [29], and 19–22% of 3321 patients enrolled into the DART study [30]. WHO guidelines have recently been revised to reflect the potential future expansion of therapeutic options (based around increased PI use) for those who fail first line therapy [31]. Thus, the problem of CSDIs is not likely to diminish in the near future.

Use of therapeutic drug monitoring is not feasible as a strategy for managing CSDIs in resource-poor settings. Practical steps that can be instituted to reduce the risk of adverse outcomes from CSDIs include integrating national treatment programmes for HIV and other diseases (with protocols that minimise drug interactions), establishing regional networks for pharmacovigilance, and improving the quality of prescribing through training and education of health care workers. Knowledge of common interactions involving antiretrovirals on a country-specific basis will allow targeted training, monitoring and protocol development. Finally, we believe that large antiretroviral programmes should undertake an audit of clinically significant drug interactions as a proxy for the quality of prescribing within that scheme.

In summary, one in three patients receiving antiretroviral therapy in Kenya were at risk of CSDIs, (which had the potential to lower HIV drug concentrations in 12% patients). Although TB medications accounted for a significant proportion of CSDIs, we identified other important interactions involving antiretrovirals such as azoles, and antimalarials. Surveys in other large cohorts are required to confirm whether these findings are generalisable to other African HIV treatment settings. Strategies need to urgently be developed to avoid important CSDIs, to identify early markers of toxicity and to manage unavoidable interactions safely in order to reduce risk of harm, and to maximize the effectiveness of mass antiretroviral deployment in Africa.
